# 5-(4-Bromo­phen­yl)-1,2,3,4-tetra­hydro­benzo[*a*]phenanthridine

**DOI:** 10.1107/S1600536809013713

**Published:** 2009-04-18

**Authors:** Heng-Shen Xie, Ai-Ling Zhang, Ling Su

**Affiliations:** a Division of Science and Technology, Xuzhou Institute of Architectural Technology, Xuzhou 221116, People’s Republic of China

## Abstract

The title compound, C_23_H_18_BrN, was synthesized by the reaction of 4-bromo­benzaldehyde, naphthalen-2-amine and cyclo­hexa­none in tetra­hydro­furan, catalyzed by iodine. The saturated six-membered ring adopts a half-chair conformation, and the four vicinal rings form a helical conformation, which results in a significant deviation from planarity for the pyridine ring. In the crystal, a weak C—H⋯π inter­action occurs, leading to inversion dimers.

## Related literature

For background on phenanthridine derivatives, see: Clement *et al.* (2005 ▶); Hazeldine *et al.* (2005 ▶); Kock *et al.* (2005 ▶); Lu *et al.* (2004 ▶); Vanquelef *et al.* (2004 ▶); Watanabe *et al.* (2003 ▶).
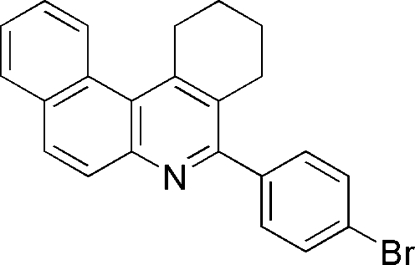


## Experimental

### 

#### Crystal data

C_23_H_18_BrN
*M* *_r_* = 388.29
Triclinic, 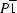

*a* = 5.660 (3) Å
*b* = 11.596 (6) Å
*c* = 13.869 (6) Å
α = 78.48 (3)°
β = 78.30 (3)°
γ = 85.15 (3)°
*V* = 872.5 (8) Å^3^
*Z* = 2
Mo *K*α radiation
μ = 2.36 mm^−1^
*T* = 296 K
0.46 × 0.22 × 0.15 mm

#### Data collection

Bruker SMART CCD diffractometer
Absorption correction: multi-scan (*SADABS*; Bruker, 2001 ▶) *T*_min_ = 0.365, *T*_max_ = 0.593 (expected range = 0.432–0.702)
11744 measured reflections
3826 independent reflections
2174 reflections with *I* > 2σ(*I*)
*R*_int_ = 0.041

#### Refinement

*R*[*F* ^2^ > 2σ(*F* ^2^)] = 0.042
*wR*(*F* ^2^) = 0.164
*S* = 1.14
3826 reflections
226 parameters
H-atom parameters constrained
Δρ_max_ = 0.94 e Å^−3^
Δρ_min_ = −1.05 e Å^−3^

### 

Data collection: *SMART* (Bruker, 2001 ▶); cell refinement: *SAINT* (Bruker, 2001 ▶); data reduction: *SAINT*; program(s) used to solve structure: *SHELXS97* (Sheldrick, 2008 ▶); program(s) used to refine structure: *SHELXL97* (Sheldrick, 2008 ▶); molecular graphics: *SHELXTL* (Sheldrick, 2008 ▶); software used to prepare material for publication: *SHELXTL*.

## Supplementary Material

Crystal structure: contains datablocks global, I. DOI: 10.1107/S1600536809013713/hb2942sup1.cif

Structure factors: contains datablocks I. DOI: 10.1107/S1600536809013713/hb2942Isup2.hkl

Additional supplementary materials:  crystallographic information; 3D view; checkCIF report

## Figures and Tables

**Table 1:** Hydrogen-bond geometry (Å, °)

*D*—H⋯*A*	*D*—H	H⋯*A*	*D*⋯*A*	*D*—H⋯*A*
C7—H7*A*⋯*Cg*^i^	0.93	2.88	3.620 (14)	137

Symmetry code: (i) 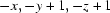. *Cg* is the centroid of the 4-bromophenyl ring.

**Fig. 1.**
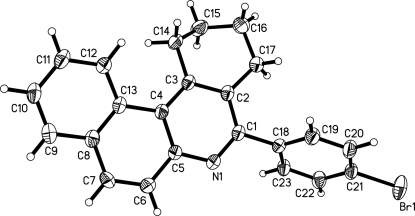
The molecular structure drawing for (I) showing 50% probability of displacement ellipsoids.

**Fig. 2.**
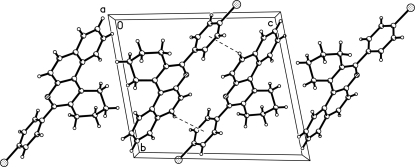
The molecular packing diagram showing the C—H···π stacking in the crystal of (I).

## supplementary crystallographic information

### Comment

Phenanthridine derivatives are well known compounds as a consequence of their pharmacological profile as an antitumor reagent (Lu, *et al.* 2004, Hazeldine, *et al.* 2005, Watanabe, *et al.* 2003). They also have been reported possessing antiviral activity (Vanquelef, *et al.* 2004), antiproliferative activity (Kock, *et al.* 2005) and cytostatic activity (Clement, *et al.* 2005), We report here the crystal structure of the title compound, (I).

The six-numbered ring (C2/C3/C14—C17) adopts a half-chair conformation: the atoms C2, C3, C14, C16 and C17 are coplanar, while the atom C15 deviates from the plane by 0.731 (6) Å; The basal plane makes a dihedral angle of 9.6 (2) ° to the coplanar pyridine ring. The dihedral angle between the pyridine ring and benzene ring (C18—C21) is 67.3 (1) °. To our surprise, the naphthalene ring is slightly distorted; with the mean deviation of fitted atoms is 0.080 Å. The largest deviation is -0.121 (2) Å for C4. Perhaps the four vicinal rings form a screw structure, which affects the plane of naphthalene ring. If the naphthalene ring is treated as two vicinal benzene rings (benzene ring (C8—C13) and benzene ring (C4—C8/C13), they make a dihedral angle of 8.6 (2) ° each other. The latter (benzene ring (C4—C8/C13) makes a dihedral angle of 7.6 (2) Å to pyridine ring. The sum of the above dihedral angles (25.8 °) of four vicinal rings is statistically equal to the dihedral angle (25.5 (2) °) between the benzene ring (C8—C13) and the plane defined by the atoms (C2/C3/C14—C17). This result also conforms that the four rings in the benza[*a*]phenanthridine moiety form a screw structure.

C—H···π stacking is present in the crystal structure of (I) (Table 2), thereby forming inversion dimers (Figure 2).

### Experimental

The title compound, (I), was prepared by the reaction of 4-bromobenzaldehyde (1 mmol, 0.185 g), naphthalen-2-amine (1 mmol, 0.143 g) and cyclohexanone (1 mmol, 0.098 g) in THF (10 ml) at 338 K catalyzed by iodine. m.p. 491–493 K. Colourless blocks of (I) were obtained by slow evaporation of an ethanol solution.

### Refinement

The H atoms were geometrically placed (C—H = 0.93–0.97Å) and refined as riding with U_iso_(H) = 1.2U_eq_(C).

### Crystal data

**Table:**

C_23_H_18_BrN	*Z* = 2
*M**_r_* = 388.29	*F*(000) = 396
Triclinic, *P*1	*D*_x_ = 1.478 Mg m^−^^3^
Hall symbol: -P 1	Melting point = 491–493 K
*a* = 5.660 (3) Å	Mo *K*α radiation, λ = 0.71073 Å
*b* = 11.596 (6) Å	Cell parameters from 3260 reflections
*c* = 13.869 (6) Å	θ = 2.6–26.1°
α = 78.48 (3)°	µ = 2.36 mm^−^^1^
β = 78.30 (3)°	*T* = 296 K
γ = 85.15 (3)°	Block, colourless
*V* = 872.5 (8) Å^3^	0.46 × 0.22 × 0.15 mm

### Data collection

**Table:**

Bruker SMART CCD diffractometer	3826 independent reflections
Radiation source: fine-focus sealed tube	2174 reflections with *I* > 2σ(*I*)
graphite	*R*_int_ = 0.041
ω scans	θ_max_ = 27.6°, θ_min_ = 1.5°
Absorption correction: multi-scan (*SADABS*; Bruker, 2001)	*h* = −7→7
*T*_min_ = 0.365, *T*_max_ = 0.593	*k* = −13→15
11744 measured reflections	*l* = −18→18

### Refinement

**Table:**

Refinement on *F*^2^	Primary atom site location: structure-invariant direct methods
Least-squares matrix: full	Secondary atom site location: difference Fourier map
*R*[*F*^2^ > 2σ(*F*^2^)] = 0.042	Hydrogen site location: inferred from neighbouring sites
*wR*(*F*^2^) = 0.164	H-atom parameters constrained
*S* = 1.14	*w* = 1/[σ^2^(*F*_o_^2^) + (0.082*P*)^2^ + 0.0233*P*] where *P* = (*F*_o_^2^ + 2*F*_c_^2^)/3
3826 reflections	(Δ/σ)_max_ = 0.001
226 parameters	Δρ_max_ = 0.94 e Å^−^^3^
0 restraints	Δρ_min_ = −1.05 e Å^−^^3^

### Special details

**Table:**

Geometry. All e.s.d.'s (except the e.s.d. in the dihedral angle between two l.s. planes) are estimated using the full covariance matrix. The cell e.s.d.'s are taken into account individually in the estimation of e.s.d.'s in distances, angles and torsion angles; correlations between e.s.d.'s in cell parameters are only used when they are defined by crystal symmetry. An approximate (isotropic) treatment of cell e.s.d.'s is used for estimating e.s.d.'s involving l.s. planes.
Refinement. Refinement of *F*^2^ against ALL reflections. The weighted *R*-factor *wR* and goodness of fit *S* are based on *F*^2^, conventional *R*-factors *R* are based on *F*, with *F* set to zero for negative *F*^2^. The threshold expression of *F*^2^ > σ(*F*^2^) is used only for calculating *R*-factors(gt) *etc*. and is not relevant to the choice of reflections for refinement. *R*-factors based on *F*^2^ are statistically about twice as large as those based on *F*, and *R*- factors based on ALL data will be even larger.

### Fractional atomic coordinates and isotropic or equivalent isotropic displacement parameters (Å^2^)

**Table:**

	*x*	*y*	*z*	*U*_iso_*/*U*_eq_	
Br1	0.54047 (10)	−0.03144 (4)	0.75111 (3)	0.0876 (3)	
N1	0.2434 (6)	0.4250 (3)	0.39876 (17)	0.0451 (7)	
C1	0.4456 (7)	0.3594 (3)	0.3874 (2)	0.0416 (8)	
C2	0.6195 (7)	0.3727 (3)	0.2984 (2)	0.0415 (8)	
C3	0.5838 (6)	0.4649 (3)	0.2203 (2)	0.0406 (8)	
C4	0.3867 (6)	0.5467 (3)	0.23571 (19)	0.0392 (8)	
C5	0.2104 (6)	0.5181 (3)	0.32479 (19)	0.0401 (8)	
C6	−0.0130 (7)	0.5835 (3)	0.3388 (2)	0.0480 (9)	
H6A	−0.1273	0.5609	0.3967	0.058*	
C7	−0.0644 (7)	0.6779 (3)	0.2703 (2)	0.0478 (9)	
H7A	−0.2165	0.7159	0.2788	0.057*	
C8	0.1163 (7)	0.7192 (3)	0.1846 (2)	0.0454 (9)	
C9	0.0690 (8)	0.8271 (3)	0.1212 (2)	0.0558 (10)	
H9A	−0.0824	0.8656	0.1317	0.067*	
C10	0.2477 (8)	0.8748 (3)	0.0442 (3)	0.0571 (11)	
H10B	0.2164	0.9446	0.0015	0.068*	
C11	0.4733 (8)	0.8188 (4)	0.0305 (2)	0.0582 (11)	
H11B	0.5952	0.8525	−0.0203	0.070*	
C12	0.5202 (7)	0.7137 (3)	0.0910 (2)	0.0486 (9)	
H12A	0.6744	0.6782	0.0805	0.058*	
C13	0.3405 (7)	0.6580 (3)	0.1688 (2)	0.0420 (9)	
C14	0.7466 (7)	0.4650 (3)	0.1191 (2)	0.0507 (9)	
H14C	0.6566	0.4976	0.0666	0.061*	
H14D	0.8805	0.5149	0.1121	0.061*	
C15	0.8432 (9)	0.3429 (4)	0.1069 (2)	0.0651 (12)	
H15C	0.9445	0.3455	0.0411	0.078*	
H15D	0.7104	0.2928	0.1124	0.078*	
C16	0.9891 (9)	0.2926 (5)	0.1872 (3)	0.0720 (13)	
H16C	1.0559	0.2149	0.1781	0.086*	
H16D	1.1221	0.3427	0.1814	0.086*	
C17	0.8312 (7)	0.2847 (4)	0.2897 (2)	0.0514 (9)	
H17A	0.7699	0.2062	0.3105	0.062*	
H17B	0.9312	0.2942	0.3365	0.062*	
C18	0.4726 (6)	0.2632 (3)	0.47485 (19)	0.0424 (8)	
C19	0.6498 (7)	0.2655 (4)	0.5296 (2)	0.0536 (10)	
H19A	0.7555	0.3268	0.5114	0.064*	
C20	0.6714 (8)	0.1771 (4)	0.6116 (2)	0.0575 (11)	
H20B	0.7932	0.1775	0.6476	0.069*	
C21	0.5097 (7)	0.0887 (3)	0.6387 (2)	0.0491 (9)	
C22	0.3329 (8)	0.0850 (3)	0.5860 (2)	0.0546 (10)	
H22A	0.2260	0.0242	0.6048	0.066*	
C23	0.3150 (7)	0.1734 (3)	0.5038 (2)	0.0498 (10)	
H23B	0.1942	0.1718	0.4676	0.060*	

### Atomic displacement parameters (Å^2^)

**Table:**

	*U*^11^	*U*^22^	*U*^33^	*U*^12^	*U*^13^	*U*^23^
Br1	0.1254 (6)	0.0660 (4)	0.0680 (3)	−0.0038 (3)	−0.0449 (3)	0.0218 (2)
N1	0.048 (2)	0.0464 (18)	0.0367 (11)	−0.0030 (16)	−0.0065 (11)	0.0014 (11)
C1	0.048 (2)	0.0384 (19)	0.0381 (13)	−0.0051 (18)	−0.0127 (13)	−0.0011 (12)
C2	0.046 (2)	0.0373 (18)	0.0410 (13)	−0.0039 (17)	−0.0133 (14)	−0.0019 (12)
C3	0.047 (2)	0.0366 (19)	0.0374 (12)	−0.0069 (17)	−0.0098 (13)	−0.0003 (12)
C4	0.042 (2)	0.0394 (19)	0.0371 (12)	−0.0066 (17)	−0.0120 (13)	−0.0022 (12)
C5	0.042 (2)	0.0390 (19)	0.0375 (13)	−0.0016 (17)	−0.0085 (13)	−0.0034 (12)
C6	0.049 (3)	0.048 (2)	0.0432 (14)	−0.0004 (19)	−0.0041 (14)	−0.0046 (14)
C7	0.044 (2)	0.043 (2)	0.0577 (17)	0.0109 (18)	−0.0132 (15)	−0.0149 (15)
C8	0.051 (3)	0.042 (2)	0.0450 (14)	−0.0058 (19)	−0.0165 (15)	−0.0041 (13)
C9	0.062 (3)	0.046 (2)	0.0621 (18)	0.004 (2)	−0.0264 (19)	−0.0037 (16)
C10	0.067 (3)	0.042 (2)	0.0589 (18)	0.000 (2)	−0.0223 (19)	0.0058 (16)
C11	0.071 (3)	0.049 (2)	0.0491 (16)	−0.011 (2)	−0.0124 (17)	0.0093 (15)
C12	0.049 (2)	0.046 (2)	0.0462 (15)	−0.0039 (18)	−0.0072 (15)	0.0003 (14)
C13	0.050 (2)	0.0373 (19)	0.0398 (13)	−0.0059 (18)	−0.0155 (14)	−0.0015 (12)
C14	0.057 (3)	0.052 (2)	0.0376 (13)	−0.0010 (19)	−0.0037 (14)	−0.0001 (13)
C15	0.079 (3)	0.061 (3)	0.0465 (16)	0.005 (2)	0.0033 (17)	−0.0091 (16)
C16	0.067 (3)	0.067 (3)	0.067 (2)	0.022 (3)	−0.001 (2)	0.000 (2)
C17	0.051 (3)	0.050 (2)	0.0490 (15)	0.0011 (19)	−0.0090 (15)	−0.0016 (15)
C18	0.045 (2)	0.043 (2)	0.0351 (12)	−0.0014 (17)	−0.0053 (12)	−0.0004 (12)
C19	0.053 (3)	0.059 (2)	0.0483 (15)	−0.014 (2)	−0.0154 (15)	0.0024 (15)
C20	0.062 (3)	0.067 (3)	0.0447 (15)	−0.006 (2)	−0.0224 (16)	−0.0004 (16)
C21	0.057 (2)	0.044 (2)	0.0434 (14)	0.0040 (18)	−0.0151 (15)	0.0017 (14)
C22	0.062 (3)	0.042 (2)	0.0572 (16)	−0.008 (2)	−0.0179 (17)	0.0056 (15)
C23	0.054 (3)	0.046 (2)	0.0487 (14)	−0.0049 (19)	−0.0195 (15)	0.0033 (14)

### Geometric parameters (Å, °)

**Table:**

Br1—C21	1.899 (3)	C12—C13	1.416 (5)
N1—C1	1.320 (5)	C12—H12A	0.9300
N1—C5	1.362 (4)	C14—C15	1.504 (6)
C1—C2	1.404 (5)	C14—H14C	0.9700
C1—C18	1.498 (4)	C14—H14D	0.9700
C2—C3	1.396 (4)	C15—C16	1.515 (6)
C2—C17	1.508 (6)	C15—H15C	0.9700
C3—C4	1.413 (5)	C15—H15D	0.9700
C3—C14	1.514 (5)	C16—C17	1.508 (5)
C4—C5	1.423 (4)	C16—H16C	0.9700
C4—C13	1.469 (4)	C16—H16D	0.9700
C5—C6	1.417 (5)	C17—H17A	0.9700
C6—C7	1.353 (5)	C17—H17B	0.9700
C6—H6A	0.9300	C18—C23	1.373 (5)
C7—C8	1.432 (5)	C18—C19	1.380 (4)
C7—H7A	0.9300	C19—C20	1.389 (5)
C8—C13	1.400 (5)	C19—H19A	0.9300
C8—C9	1.418 (5)	C20—C21	1.376 (5)
C9—C10	1.374 (6)	C20—H20B	0.9300
C9—H9A	0.9300	C21—C22	1.361 (5)
C10—C11	1.378 (6)	C22—C23	1.387 (4)
C10—H10B	0.9300	C22—H22A	0.9300
C11—C12	1.373 (5)	C23—H23B	0.9300
C11—H11B	0.9300		
			
C1—N1—C5	118.9 (3)	C15—C14—H14C	109.3
N1—C1—C2	123.4 (3)	C3—C14—H14C	109.3
N1—C1—C18	115.2 (3)	C15—C14—H14D	109.3
C2—C1—C18	121.3 (3)	C3—C14—H14D	109.3
C3—C2—C1	118.2 (4)	H14C—C14—H14D	108.0
C3—C2—C17	122.8 (3)	C14—C15—C16	109.5 (3)
C1—C2—C17	119.0 (3)	C14—C15—H15C	109.8
C2—C3—C4	119.4 (3)	C16—C15—H15C	109.8
C2—C3—C14	117.4 (3)	C14—C15—H15D	109.8
C4—C3—C14	122.9 (3)	C16—C15—H15D	109.8
C3—C4—C5	117.0 (3)	H15C—C15—H15D	108.2
C3—C4—C13	127.2 (3)	C17—C16—C15	110.2 (4)
C5—C4—C13	115.9 (3)	C17—C16—H16C	109.6
N1—C5—C6	116.8 (3)	C15—C16—H16C	109.6
N1—C5—C4	122.1 (3)	C17—C16—H16D	109.6
C6—C5—C4	121.1 (3)	C15—C16—H16D	109.6
C7—C6—C5	121.7 (3)	H16C—C16—H16D	108.1
C7—C6—H6A	119.2	C2—C17—C16	116.1 (3)
C5—C6—H6A	119.2	C2—C17—H17A	108.3
C6—C7—C8	119.8 (4)	C16—C17—H17A	108.3
C6—C7—H7A	120.1	C2—C17—H17B	108.3
C8—C7—H7A	120.1	C16—C17—H17B	108.3
C13—C8—C9	121.3 (3)	H17A—C17—H17B	107.4
C13—C8—C7	120.1 (3)	C23—C18—C19	119.0 (3)
C9—C8—C7	118.5 (4)	C23—C18—C1	120.2 (3)
C10—C9—C8	119.7 (4)	C19—C18—C1	120.8 (3)
C10—C9—H9A	120.1	C18—C19—C20	120.5 (3)
C8—C9—H9A	120.1	C18—C19—H19A	119.8
C9—C10—C11	119.8 (3)	C20—C19—H19A	119.8
C9—C10—H10B	120.1	C21—C20—C19	118.9 (3)
C11—C10—H10B	120.1	C21—C20—H20B	120.6
C12—C11—C10	120.8 (4)	C19—C20—H20B	120.6
C12—C11—H11B	119.6	C22—C21—C20	121.6 (3)
C10—C11—H11B	119.6	C22—C21—Br1	119.9 (3)
C11—C12—C13	121.9 (4)	C20—C21—Br1	118.5 (2)
C11—C12—H12A	119.1	C21—C22—C23	118.8 (3)
C13—C12—H12A	119.1	C21—C22—H22A	120.6
C8—C13—C12	116.3 (3)	C23—C22—H22A	120.6
C8—C13—C4	120.5 (3)	C18—C23—C22	121.2 (3)
C12—C13—C4	123.0 (3)	C18—C23—H23B	119.4
C15—C14—C3	111.6 (3)	C22—C23—H23B	119.4
			
C5—N1—C1—C2	−6.8 (4)	C7—C8—C13—C12	−171.1 (3)
C5—N1—C1—C18	176.7 (3)	C9—C8—C13—C4	179.6 (3)
N1—C1—C2—C3	4.2 (5)	C7—C8—C13—C4	4.4 (4)
C18—C1—C2—C3	−179.5 (3)	C11—C12—C13—C8	−3.6 (5)
N1—C1—C2—C17	−173.5 (3)	C11—C12—C13—C4	−178.9 (3)
C18—C1—C2—C17	2.8 (4)	C3—C4—C13—C8	167.9 (3)
C1—C2—C3—C4	5.1 (4)	C5—C4—C13—C8	−10.9 (4)
C17—C2—C3—C4	−177.3 (3)	C3—C4—C13—C12	−16.9 (5)
C1—C2—C3—C14	−169.1 (3)	C5—C4—C13—C12	164.3 (3)
C17—C2—C3—C14	8.5 (5)	C2—C3—C14—C15	25.2 (4)
C2—C3—C4—C5	−11.0 (4)	C4—C3—C14—C15	−148.8 (3)
C14—C3—C4—C5	162.9 (3)	C3—C14—C15—C16	−60.4 (5)
C2—C3—C4—C13	170.1 (3)	C14—C15—C16—C17	61.1 (5)
C14—C3—C4—C13	−16.0 (5)	C3—C2—C17—C16	−6.9 (5)
C1—N1—C5—C6	177.9 (3)	C1—C2—C17—C16	170.7 (3)
C1—N1—C5—C4	0.1 (4)	C15—C16—C17—C2	−27.8 (6)
C3—C4—C5—N1	8.7 (4)	N1—C1—C18—C23	63.7 (4)
C13—C4—C5—N1	−172.3 (3)	C2—C1—C18—C23	−113.0 (4)
C3—C4—C5—C6	−169.0 (3)	N1—C1—C18—C19	−114.0 (4)
C13—C4—C5—C6	10.0 (4)	C2—C1—C18—C19	69.3 (5)
N1—C5—C6—C7	179.7 (3)	C23—C18—C19—C20	1.2 (6)
C4—C5—C6—C7	−2.5 (5)	C1—C18—C19—C20	179.0 (3)
C5—C6—C7—C8	−4.7 (5)	C18—C19—C20—C21	−1.5 (6)
C6—C7—C8—C13	3.6 (5)	C19—C20—C21—C22	1.3 (6)
C6—C7—C8—C9	−171.7 (3)	C19—C20—C21—Br1	−179.8 (3)
C13—C8—C9—C10	−1.6 (5)	C20—C21—C22—C23	−0.7 (6)
C7—C8—C9—C10	173.6 (3)	Br1—C21—C22—C23	−179.6 (3)
C8—C9—C10—C11	−1.5 (5)	C19—C18—C23—C22	−0.7 (6)
C9—C10—C11—C12	2.0 (6)	C1—C18—C23—C22	−178.4 (3)
C10—C11—C12—C13	0.6 (5)	C21—C22—C23—C18	0.4 (6)
C9—C8—C13—C12	4.0 (4)		

### Hydrogen-bond geometry (Å, °)

**Table:**

*D*—H···*A*	*D*—H	H···*A*	*D*···*A*	*D*—H···*A*
C7—H7A···Cg^i^	0.93	2.88	3.620 (14)	137

Symmetry codes: (i) −*x*, −*y*+1, −*z*+1.
